# Associations of systemic inflammatory regulators with CKD and kidney function: evidence from the bidirectional mendelian randomization study

**DOI:** 10.1186/s12882-024-03590-2

**Published:** 2024-05-10

**Authors:** Hailang Liu, Wei Xiang, Wei Wu, Gaofeng Zhou, Jingdong Yuan

**Affiliations:** Department of Urology, Wuhan Integrated TCM & Western Medicine Hospital, Tongji Medical College, Huazhong University of Science and Technology, Wuhan No.1 Hospital, Wuhan, China

**Keywords:** Chronic kidney disease, Kidney function, Mendelian randomization, Inflammation, Cytokines

## Abstract

**Background:**

Previous observational studies have reported that systemic inflammatory regulators are related to the development of chronic kidney disease (CKD); however, whether these associations are causal remains unclear. The current study aimed to investigate the potential causal relationships between systemic inflammatory regulators and CKD and kidney function.

**Method:**

We performed bidirectional two-sample Mendelian randomization (MR) analyses to infer the underlying causal associations between 41 systemic inflammatory regulators and CKD and kidney function. The inverse-variance weighting (IVW) test was used as the primary analysis method. In addition, sensitivity analyses were executed via the Mendelian randomization pleiotropy residual sum and outlier (MR-PRESSO) test and the weighted median test.

**Results:**

The findings revealed 12 suggestive associations between 11 genetically predicted systemic inflammatory regulators and CKD or kidney function in the forward analyses, including 4 for CKD, 3 for blood urea nitrogen (BUN), 4 for eGFRcrea and 1 for eGFRcys. In the other direction, we identified 6 significant causal associations, including CKD with granulocyte-colony stimulating factor (GCSF) (IVW β = 0.145; 95% CI, 0.042 to 0.248; *P* = 0.006), CKD with stem cell factor (SCF) (IVW β = 0.228; 95% CI, 0.133 to 0.323; *P* = 2.40 × 10^− 6^), eGFRcrea with SCF (IVW β =-2.90; 95% CI, -3.934 to -1.867; *P* = 3.76 × 10^− 8^), eGFRcys with GCSF (IVW β =-1.382; 95% CI, -2.404 to -0.361; *P* = 0.008), eGFRcys with interferon gamma (IFNg) (IVW β =-1.339; 95% CI, -2.313 to -0.366; *P* = 0.007) and eGFRcys with vascular endothelial growth factor (VEGF) (IVW β =-1.709; 95% CI, -2.720 to -0.699; *P* = 9.13 × 10^− 4^).

**Conclusions:**

Our findings support causal links between systemic inflammatory regulators and CKD or kidney function both in the forward and reverse MR analyses.

**Supplementary Information:**

The online version contains supplementary material available at 10.1186/s12882-024-03590-2.

## Introduction

Chronic kidney disease (CKD) is a progressive disease with high morbidity and mortality, affecting > 10% of the adult population worldwide, especially in people with diabetes and hypertension [1]. The global burden of CKD is substantial and growing, and it’s reported that CKD results in 1.2 million deaths and 28.0 million years of life lost each year [2]. Although treatment has been demonstrated to slow progression, CKD usually gets worse over time at different paces [3]. In the end stage, CKD can progress to kidney failure and early cardiovascular disease [1, 3]. Kidney failure, also called end-stage renal disease (ESRD), is fatal without dialysis or a kidney transplant [1, 3]. CKD is often associated with higher comorbidity and poor quality of life, especially in the stage of ESRD [4]. Patients with CKD may experience higher rates of hospitalization due to the high prevalence of cardiovascular, infectious, hormonal, nutritional and psychological disorders [5]. Owing to its few or nonspecific clinical symptoms, CKD is scarcely diagnosed in initial stages, and once progressed, the damage to kidneys is unfortunately irreversible [6]. Besides the huge economic burden that CKD’s treatment involves, how to achieve satisfied long-term outcomes in these patients is a major challenge all over the world. Therefore, identifying the potential causal factors for CKD and the direction of their impact could be beneficial for informing prevention strategies.

Persistent, low-grade systemic inflammation, usually characterized by persistent, low to moderate levels of circulating inflammation markers, is now considered a hallmark feature of CKD [6, 7]. Accumulating evidence has demonstrated that cytokines, as crucial inflammatory regulators, may contribute to the onset and progression of CKD, and are associated with many complications during CKD, such as coronary artery calcification, malnutrition, heart failure and atherosclerosis [6–8]. Important markers of inflammation in CKD include tumor necrosis factor alpha (TNFa), adipokines, C-reactive protein (CRP), interleukin-18 (IL-18), interleukin-6 (IL-6), interleukin-1 (IL-1), adhesion molecules and the CD40 ligand [7–9]. However, it’s worth noting that the associations of the pathogenesis of CKD with inflammation markers are mainly derived from conventional observational studies and are thus susceptible to biases, such as reverse causality, small sample size, and confounding effects. Due to the existence of these biases and the inevitable heterogeneity among different studies, it is sometimes difficult to reach a robust, convincing conclusion on the association between inflammation markers and CKD according the results of previous studies. It is critical to investigate the putative causal role of systemic inflammatory regulators on CKD and kidney function, and vice versa.

The Mendelian randomization (MR) design is an emerging genetic method that can strengthen causal inference regarding an exposure-outcome association by leveraging genetic variants as instrumental variables for exposure [10]. By utilizing genetic instruments as proxies for environmental exposures, MR analysis circumvents many of the confounding factors and biases inherent in traditional observational studies. This methodological framework is grounded in the principle of Mendel’s laws of inheritance, which postulate that alleles are randomly assigned during gamete formation, thereby creating a natural randomization that mimics the design of a randomized controlled trial [11, 12]. Moreover, this method can theoretically diminish reverse causality because the genesis and development of disease cannot modify the germline genotype [11, 12].

Herein, we conducted a two sample MR investigation to explore the potential causal associations of genetic liability for circulating inflammation markers with CKD risk and kidney function based on the most recent and largest genome-wide association studies (GWAS). In addition, we also assessed the MR effects of CKD and kidney function on systemic inflammatory regulators.

## Methods

### MR design

As a genetic variant is usually deemed a proxy for a risk factor in an MR design, the choice of a genetic instrument variable (IV) is particularly important for a successful MR study. MR requires three basic IV assumptions to validate a genetic variant as valid IVs for causal inference: (1) the genetic variant should be robustly associated with the exposure; (2) the genetic variant is not related to potential confounders of the exposure-outcome association; and (3) the genetic variant should have no effect on the outcome other than through the exposure.

### Data sources

We extracted GWAS summary statistics of 41 systemic inflammatory regulators processed by Ahola-Olli et al. [13], with a sample size of 8293 Finnish individuals from three cohorts: FINRISK1997, FINRISK2002 and the Cardiovascular Risk in Young Finns Study (YFS). GWAS was adjusted for sex, age, body mass index and the first ten genetic principal components.

CKD is defined as an estimated glomerular filtration rate (eGFR) below 60 mL min^− 1^ (1.73m^2^)^−1^. Kidney function-related outcomes included eGFRcys (calculated using the Chronic Kidney Disease Epidemiology Collaboration cystatin C equation), eGFRcrea (calculated using the Chronic Kidney Disease Epidemiology Collaboration creatinine equation), blood urea nitrogen (BUN) and CKD. We drew summary statistics of GWASs associated with CKD and kidney function from the CKDGen consortium in the public domain (https://ckdgen.imbi.unifreiburg.de/), which is the current largest independent GWAS meta-analysis results for kidney function traits [14, 15]. The single-nucleotide polymorphisms (SNPs) for CKD were chosen from a meta-analysis of GWAS with a total of 480,698 participants of European ancestry [14]. The SNPs for kidney function came from a meta-analysis of GWAS with 1,201,909 individuals (765,348 participants from CKDGen consortium and 436,581 participants from UK Biobank) [15].

### Genetic instrument selection

To examine the MR effects of 41 systemic inflammatory regulators on CKD and kidney function, SNPs associated with exposure were obtained at the genome-wide significance level (*P* < 5 × 10^− 8^) at first (Supplementary Table S2). We found that only 9 of the systemic inflammatory regulators had no less than 3 significant SNPs at the genome-wide significance level. We then set the genome-wide significance level at *P* < 5 × 10^− 6^ (Supplementary Table S3) to select associated SNPs. SNP-related linkage disequilibrium (LD) was estimated based on the 1000 Genomes European reference panel [16]. In order to exclude SNPs in LD, a strict cut-off of r^2^ < 0.01 and a window of 10,000 kb were used for clumping. The SNP with the smallest *P* value for the genetic association for every risk factor was attained. Those SNPs which met the aforementioned criteria were used as instrumental variables (IVs) in MR analysis. Given that the F statistic is mainly used to evaluate instrument strength, we first calculated F statistic for each selected SNP using the formula [17]: F = R^2^ (N − k−1)/k(1 − R^2^ ) (N, the sample size of GWAS for exposure; k, the number of instruments). The R^2^ represented the proportion of variance of genetic instruments on exposure and the R^2^ of a single IV was calculated using the formula [18]: R^2^ = β^2^/(β^2^ + SE×N)( β, genetic effect size of selected SNPs of exposure; SE, standard error of genetic effect size of selected SNPs). To estimate the F statistic for each systemic inflammatory regulator, the R^2^ in the formula for computing was the sum of R^2^ of each genetic variant [18]. Generally, an F statistic of ≥ 10 suggests a relatively low risk of bias caused by weak IVs in MR analysis [19]. Subsequently, we harmonized the effects of SNPs on exposure and outcome in order to guarantee that the β values were signed to the same alleles and remove palindromic SNPs with ambiguous minor allele frequency > 0.45 and < 0.55 [20]. Additionally, we also excluded outlier pleiotropic SNPs using Mendelian Randomization pleiotropy residual sum and outlier (MR-PRESSO) method with a *P* value threshold of 0.05 [21]. Eventually, the remaining SNPs which met the assumption and selection criteria were used to construct the genetic IVs for systemic inflammatory regulators to perform MR analysis.

We performed the reverse MR analysis to explore the MR effects of CKD and kidney function on the concentrations of 41 systemic inflammatory regulators and the *P* value threshold of 5 × 10^− 8^ for selecting IVs was used. The subsequent selection steps for constructing the genetic IVs were similar to those for the 41 systemic inflammatory markers. All exposures had 3 or more valid IVs and are presented in Supplementary Table S4.

### Statistical analysis

We used the method of inverse-variance weighting (IVW) with random effects to estimate MR associations between genetic liability to systemic inflammatory regulators and the risk of CKD and kidney function. IVW method assumes that all SNPs are valid instrumental variables and that the estimates can be interpreted to reflect the total effect of the exposure [22, 23]. It was the primary analysis used to assess causality in this study. Given that the IVW approach only generates an unbiased estimate under the MR assumptions that there is no invalid instrument and horizontal pleiotropy, two sensitivity analysis methods, Mendelian Randomization pleiotropy residual sum and outlier (MR-PRESSO) [21] and the weighted median [24], were carried out to examine the robustness of the results and detect horizontal pleiotropy, if any. The MR-Egger method generates a causal effect estimate based on the slope coefficient from Egger regression, which gives a more robust and reliable causal effect estimate even if all of the IVs are invalid [23]. MR-Egger regression analysis can detect and correct for directional pleiotropy, whereas it compromises power. The weighted median method specifies that the MR estimates are robust even when up to 50% of the information comes from invalid instrumental variables [24]. The heterogeneity of independent SNP effects was assessed by Cochran’s Q statistics of the IVW and MR-Egger estimates; a *P* value of < 0.05 would be regarded as indicative of significant heterogeneity. We performed MR-PRESSO analysis to identify possible outliers and generate estimates corrected for outliers (a *P* value of < 0.05 was considered as an outlier) [21]. In addition, the MR-PRESSO Global test and intercept of the MR-Egger test were executed to determine the presence of horizontal and directional pleiotropy (*P* < 0.05), respectively. Odds ratios (ORs) and corresponding confidence intervals (CIs) of CKD and kidney function were scaled to a one-standard deviation (SD) increase in systemic inflammatory regulators.

To examine the causal association between systemic inflammatory regulators and CKD, bidirectional MR analyses were conducted in the present study. Forward MR analyses were executed to investigate the MR effects of systemic inflammatory regulators on CKD and kidney function. Afterwards, reverse MR analyses were executed to investigate the MR effects of CKD and kidney function on the level of 41 systemic inflammatory regulators.

All analyses were conducted using R version 4.2.1 and MR analysis was performed using the TwoSampleMR, Mendelian Randomization and MR-PRESSO packages in the R software. To address the issue of multiple testing, a Bonferroni-corrected significance threshold was applied. Associations between the 41 systemic inflammatory regulators and outcomes were considered statistically significant when the *P* value was less than a Bonferroni-corrected significance threshold. *P* values of associations between Bonferroni-corrected significance level and 0.05 were considered as suggestive of a potential association.

## Results

**Fig. 1:**
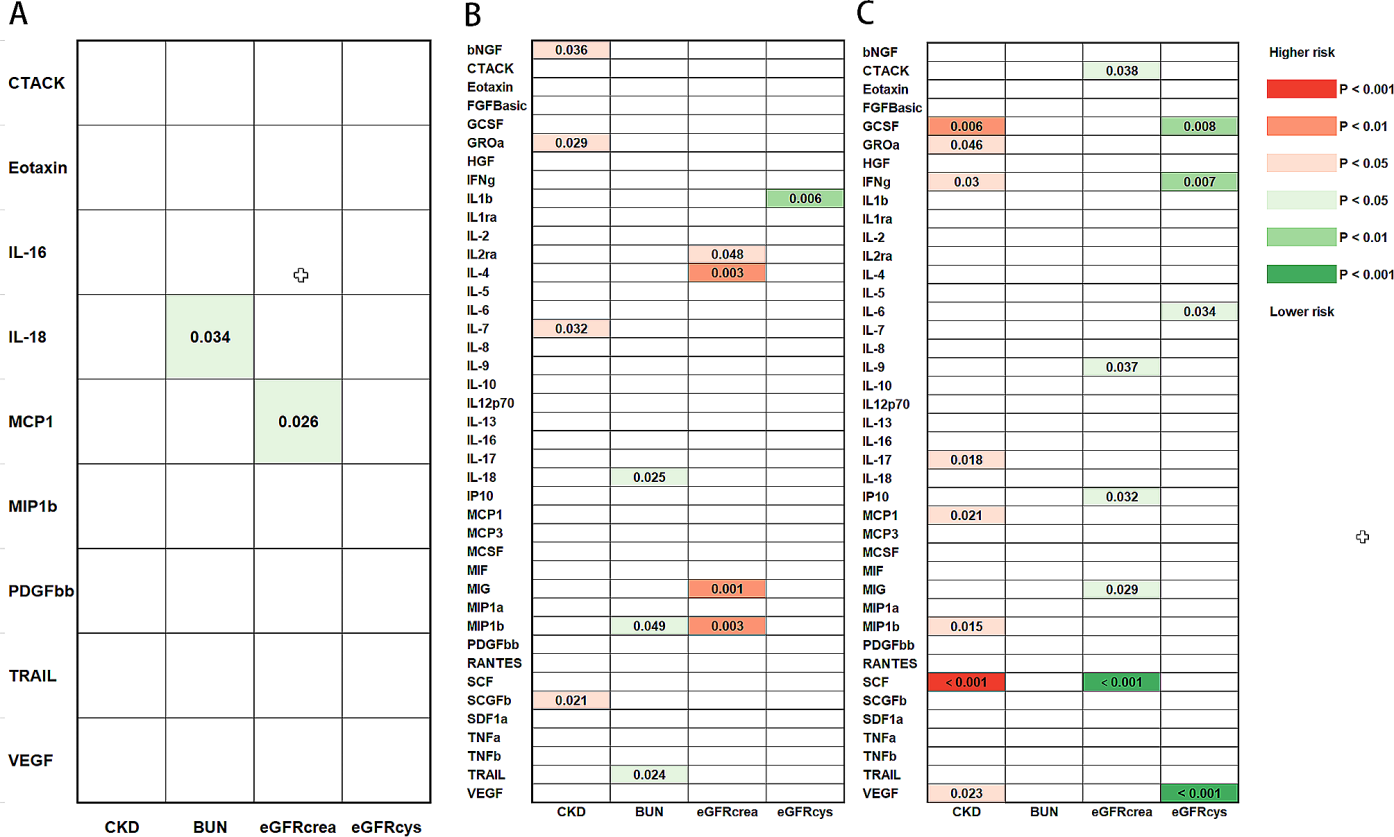
The causal relationships among systemic inflammatory regulators and CKD and kidney function. Shown are the *P* values derived from the IVW analyses results. (**A**) MR effects of systemic inflammatory regulators on CKD and kidney function (SNPs reaching *P* < 5 × 10^− 8^ used as IVs). (**B**) MR effects of systemic inflammatory regulators on CKD and kidney function (SNPs reaching *P* < 5 × 10^− 6^ used as IVs). (**C**) MR effects of CKD and kidney function on the concentrations of systemic inflammatory regulators (SNPs for CKD reaching *P* < 5 × 10^− 8^ used as IVs)

The F statistics for IVs are presented in Supplementary Table S2, Table S3 and Table S4. All F-statistics were above the threshold of 10, suggesting the absence of weak instrument bias in MR analyses in this study. IVW test, the primary analysis method in this study, was used to investigate the causal relationship between systemic inflammatory regulators and CKD and kidney function. Figure 1 displays the overall associations by the IVW test.

### The MR effects of genetically predicted systemic inflammatory regulators on the risk of CKD and kidney function

**Fig. 2:**
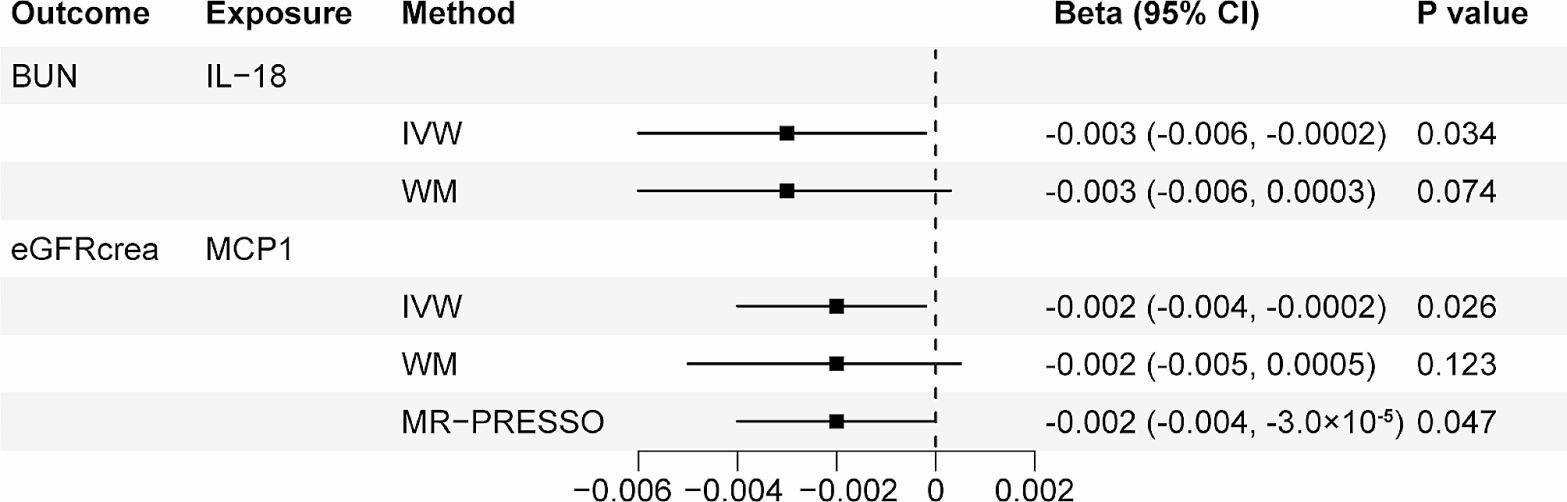
Associations of systemic inflammatory regulators with CKD and kidney function. Shown are relationships with *P* < 0.05 in the IVW models. SNPs reaching *P* < 5 × 10^− 8^ were used as IVs

When the genome-wide significance level of exposure was set as *P* < 5 × 10^− 8^, the Bonferroni-corrected threshold was set as 0.05/9 (0.006) after multiple testing correction. We noted a suggestive association between genetically predicted high interleukin 18 (IL-18) and decreased BUN (IVW β =-0.003; 95% confidence interval (CI), -0.006 to -0.0002; *P* = 0.034). We also noted a suggestive association between high monocyte chemotactic protein-1 (MCP 1) and decreased eGFRcrea (IVW β =-0.002; 95% CI, -0.004 to -0.0002; *P* = 0.026). However, the associations of IL-18 with BUN (IVW β =-0.003; 95% CI, -0.006 to 0.0003; *P* = 0.074) and MCP 1 with eGFRcrea (IVW β =-0.002; 95% CI, -0.005 to 0.0005; *P* = 0.123) were not consistent in the weighted median analysis. MR-Egger intercept analysis showed no evidence of directional pleiotropy for both IL-18 and MCP 1 (*P* = 0.51 and *P* = 0.671, respectively). Additionally, heterogeneity was not detected for both IL-18 and MCP 1 (Cochran *P* value = 0.509 and Cochran *P* value = 0.702, respectively) (Fig. 2 and Supplementary Table S5).

**Fig. 3:**
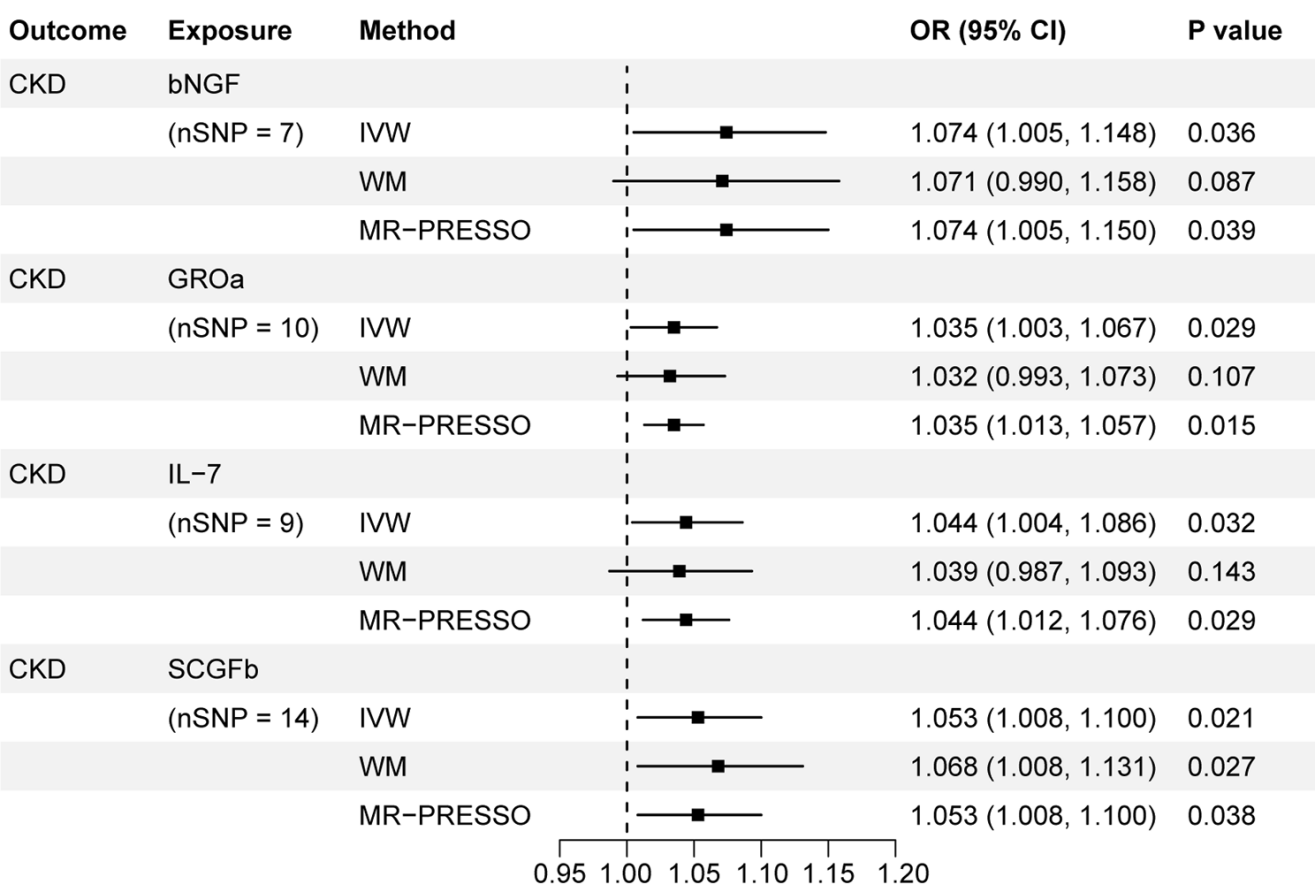
Associations of systemic inflammatory regulators with CKD. Shown are relationships with *P* < 0.05 in the IVW models. SNPs reaching *P* < 5 × 10^− 6^ were used as IVs

**Fig. 4:**
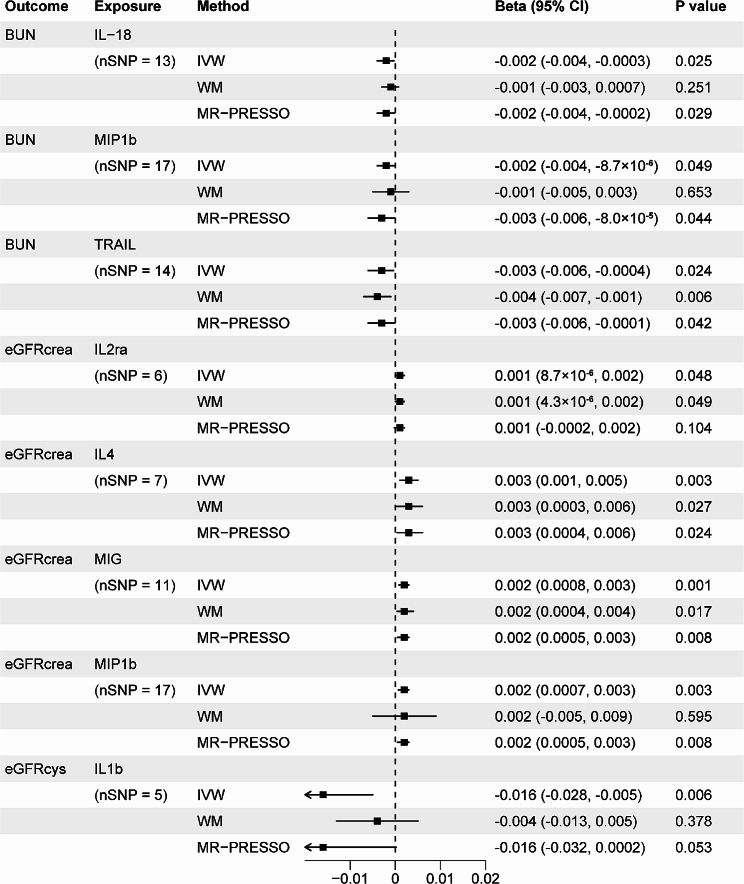
Associations of systemic inflammatory regulators with kidney function. Shown are relationships with *P* < 0.05 in the IVW models. SNPs reaching *P* < 5 × 10^− 6^ were used as IVs

When the genome-wide significance level of exposure was set as *P* < 5 × 10^− 6^, the Bonferroni-corrected threshold was set as 0.05/41 (0.001) after multiple testing correction. All 41 systemic inflammatory regulators had at least 3 available IVs. We found a total of 12 suggestive associations involving 11 systemic inflammatory regulators, indicating that none of these associations were statistically significant at the level of Bonferroni-corrected *P* value (0.001). Higher genetically predicted beta-nerve growth factor (bNGF) (IVW OR = 1.074; 95% CI, 1.005–1.148; *P* = 0.036), growth-regulated protein alpha (GROa) (IVW OR = 1.035; 95% CI, 1.003–1.067; *P* = 0.029), IL-7 (IVW OR = 1.044; 95% CI, 1.004–1.086; *P* = 0.032) and stem cell growth factor beta (SCGFb) (IVW OR = 1.053; 95% CI, 1.008-1.100; *P* = 0.021) were associated with increased risk of CKD. The MR effect estimate was similar in size in weighted median analysis of SCGFb with CKD (OR = 1.068; 95% CI, 1.008–1.131; *P* = 0.027). Genetically high IL-18 (IVW β =-0.002; 95% CI, -0.004 to -0.0003; *P* = 0.025), macrophage inflammatory protein 1b (MIP1b) (IVW β =-0.002; 95% CI, -0.004 to -8.7 × 10^− 6^; *P* = 0.049) and TNF-related apoptosis-inducing ligand (TRAIL) (IVW β =-0.003; 95% CI, -0.006 to -0.0004; *P* = 0.024) were inversely associated with BUN, supporting the protective effects of these systemic inflammatory regulators on kidney function. The univariable MR estimates were nominally significant for the effect of genetically high IL-2ra (IVW β = 0.001; 95% CI, 8.7 × 10^− 6^ to 0.002; *P* = 0.048) or IL-4 (IVW β = 0.003; 95% CI, 0.001 to 0.005; *P* = 0.003) or monokine induced by gamma interferon (MIG) (IVW β = 0.002; 95% CI, 0.0008 to 0.003; *P* = 0.001) or MIP1b (IVW β = 0.002; 95% CI, 0.0007 to 0.003; *P* = 0.003) with eGFRcrea, also indicating the protective role of these systemic inflammatory regulators on kidney function. However, genetically predicted higher IL-1b (IVW β =-0.016; 95% CI, -0.028 to -0.005; *P* = 0.006) was found to be negatively associated with eGFRcys. Based on the results of MR-Egger intercept analyses, we find no evidence of directional pleiotropy in all suggestive associations except MIP1b with eGFRcrea (*P* = 0.026) and TRAIL with BUN (*P* = 0.008). Moreover, heterogeneity was not detected for all suggestive associations except IL-1b with eGFRcys (Cochran *P* value < 0.001) according to Cochran’s Q values derived from MR-Egger and IVW tests (Figs. 3 and 4, and Supplementary Table S6).

### The MR effects of genetically predicted CKD and kidney function on systemic inflammatory regulators level

**Fig. 5:**
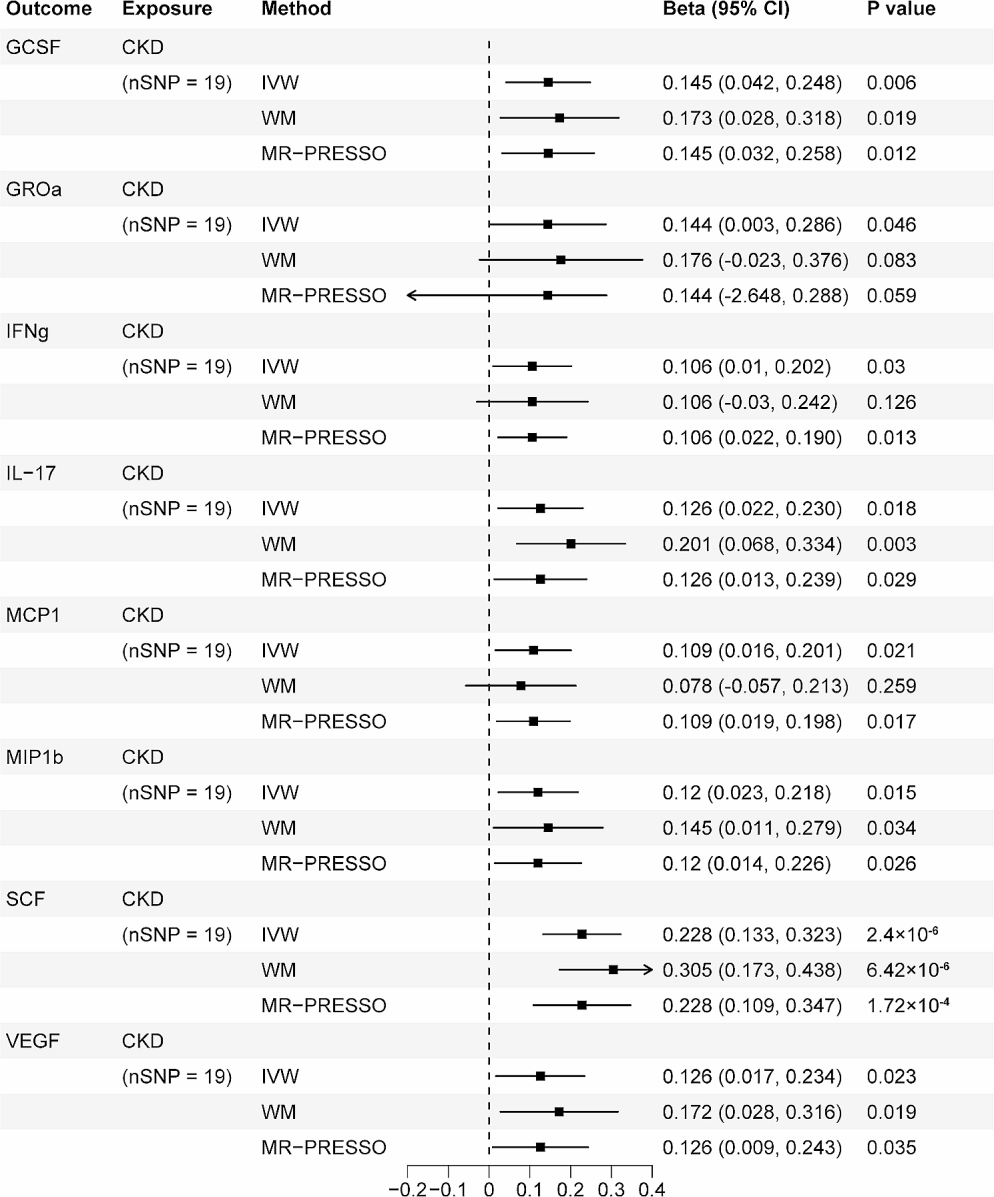
Associations of CKD with systemic inflammatory regulators. Shown are relationships with *P* < 0.05 in the IVW models. SNPs for CKD reaching *P* < 5 × 10^− 8^ were used as IVs

**Fig. 6:**
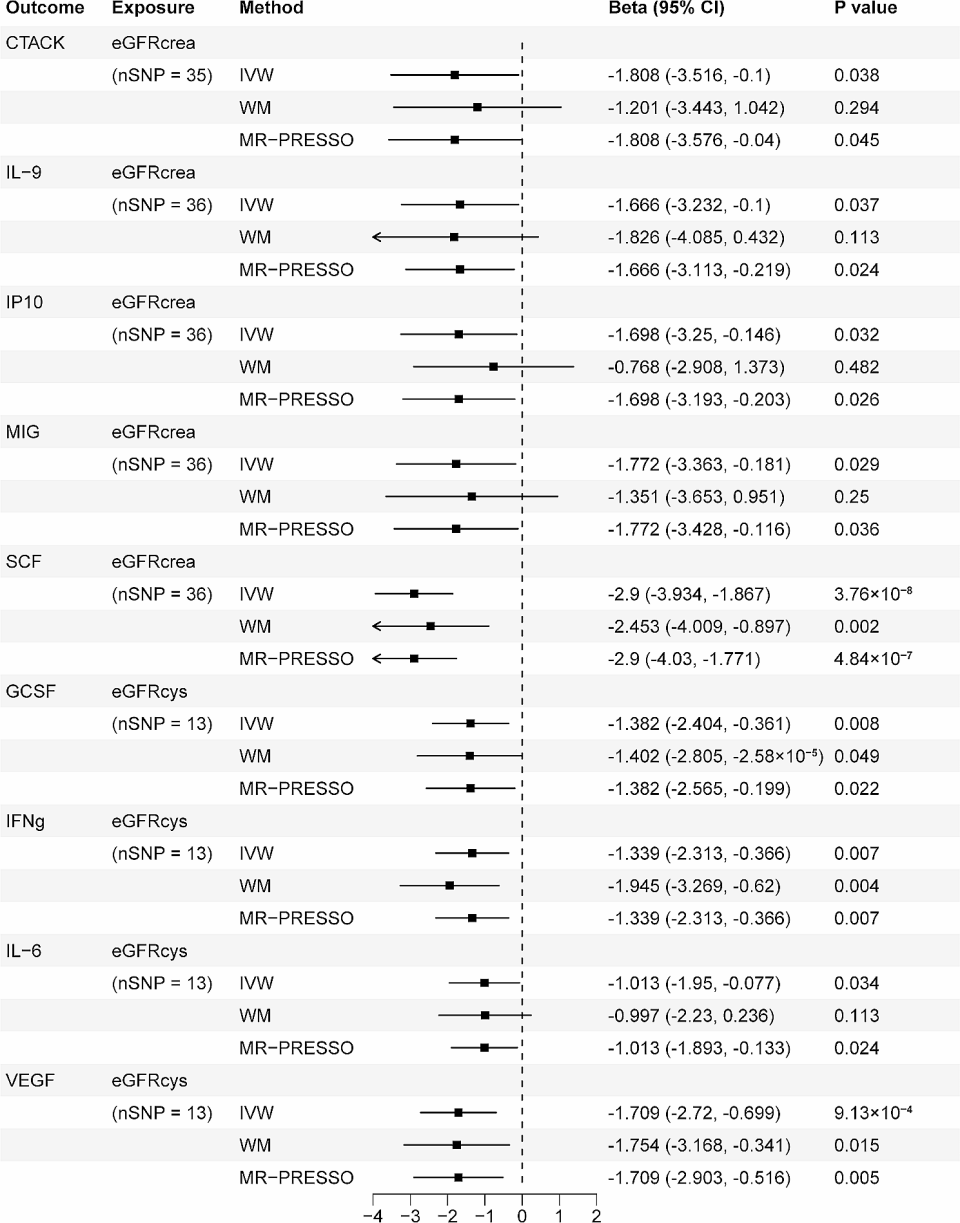
Associations of kidney function with systemic inflammatory regulators. Shown are relationships with *P* < 0.05 in the IVW models. SNPs for CKD reaching *P* < 5 × 10^− 8^ were used as IVs

In the opposite direction, with genetic liability for CKD and kidney function as exposures, we executed MR analyses to examine the causal relationship between CKD and kidney function and all the 41 systemic inflammatory regulators. We identified 6 statistically significant and 11 suggestive associations in total. Those statistically significant associations included CKD with granulocyte-colony stimulating factor (GCSF) (IVW β = 0.145; 95% CI, 0.042 to 0.248; *P* = 0.006), CKD with stem cell factor (SCF) (IVW β = 0.228; 95% CI, 0.133 to 0.323; *P* = 2.40 × 10^− 6^), eGFRcrea with SCF (IVW β =-2.90; 95% CI, -3.934 to -1.867; *P* = 3.76 × 10^− 8^), eGFRcys with GCSF (IVW β =-1.382; 95% CI, -2.404 to -0.361; *P* = 0.008), eGFRcys with interferon gamma (IFNg) (IVW β =-1.339; 95% CI, -2.313 to -0.366; *P* = 0.007) and eGFRcys with vascular endothelial growth factor (VEGF) (IVW β =-1.709; 95% CI, -2.720 to -0.699; *P* = 9.13 × 10^− 4^), indicating that all *P* values were less than the Bonferroni-corrected significance threshold (0.05/4 = 0.0125). The MR effect estimates of significant associations from the weighted median and MR-PRESSO methods were similar with those from the corresponding IVW models in size and direction, except eGFRcys with GCSF (*P* = 0.0502) in the weighted median analysis. This demonstrated that the results of our primary analysis were robust. Moreover, using the MR-Egger intercept and the MR-PRESSO Global test, we did not find the evidence of horizontal and directional pleiotropy among the significant associations (all *P* > 0.05). With regard to heterogeneity, all Cochran *P* values for significant associations based on MR-Egger and IVW tests were larger than 0.05, suggesting that there was no obvious heterogeneity (Figs. 5 and 6, and Supplementary Table S7).

## Discussion

In this study, we performed bidirectional MR analyses to investigate the causal relationship between CKD and kidney function and the 41 systemic inflammatory regulators. Our findings revealed that genetically predicted levels of 11 systemic inflammatory regulators showed potential evidence of MR effects on CKD and kidney function in the forward MR analyses, including bNGF, GROa, IL-7 and SCGFb for CKD, IL-18, MIP1b and TRAIL for BUN, IL-2ra, IL-4, MIG and MIP1b for eGFRcrea and IL-1b for eGFRcys. Meanwhile, in the reverse MR analyses, genetically liability to CKD and kidney function was significantly associated with the concentrations of 4 systemic inflammatory regulators, including CKD, eGFRcys with GCSF, CKD, eGFRcrea with SCF, eGFRcys with IFNg and eGFRcys with VEGF. In addition, these associations were robust to different methods in general.

Remarkably, IL-1b, was found to have a suggestive MR effect (IVW OR = 0.984; 95% CI, 0.972–0.995; *P* = 0.006) on eGFRcys. IL-1b is a member of the IL-1 family which consists of 11 members, 4 inflammatory antagonists (IL-1ra, IL-36ra, IL-37 and IL-38) and 7 pro-inflammatory agonists (IL-1a, IL-1b, IL-18, IL-36a, IL-36b, IL-36γ and IL-33) [25, 26]. It is well recognized that the main function of IL-1b is to result in immune activation and fever by binding to IL-1 receptor and to regulate the generation of T helper 17 cells [25]. Nevertheless, there seemed no consensus on the relationship between IL-1b and CKD or kidney function to date. Gupta J et al. [27] concluded that plasma level of IL-1b was negatively associated with eGFR. While in the study conducted by Pruijm M et al. [28], a significant association between eGFRcrea and IL-1b was not detected. Data from the CRIC Study revealed that increased plasma level of IL-1b was not significantly related to rapid loss of kidney function in patients with CKD [29]. In contrast, our study illustrated that genetically predicted high level of IL-1b was inversely associated with eGFRcys. The suggestive MR effect of IL-18 on BUN (IVW OR = 0.998; 95% CI, 0.996–0.999; *P* = 0.025) was also identified in the forward MR analyses. Likewise, IL-18, a member of the IL-1 family of cytokines, has been corroborated to be constitutively present in nearly all cells of healthy humans and animals [30]. A previous study reported that IL-18 blockade could ameliorate renal fibrosis and obstruction-induced epithelial-mesenchymal transition to protect against CKD in mice [31]. Similarly, a recent study also demonstrated that IL-18 deficiency mitigated the progression from AKI to CKD in mice [32]. Nonetheless, considering that a genetically predicted high level of IL-18 was inversely associated with the concentrations of BUN, IL-18 seemed to be a renoprotective role in the progression of CKD according to the results of this study. Discrepancy between our results and the previous two articles regarding the effect of IL-18 on CKD may be attributed to the fact that their findings were derived from animal models while ours were from humans. Of note, the pyrin domain-containing protein 3 (NLRP3) inflammasome, an important component of innate immunity and contributor to the pathology of many human diseases, can facilitate the maturation and caspase 1-dependent production of IL-1b and IL-18 and mature IL-1b and IL-18 can further activate inflammatory cascades and participate in the pathogenesis of organs, such as the kidney [33–35]. Numerous studies have demonstrated that both IL-6 and TNFa play an important role in the onset and progression of CKD [7, 29, 35, 36]. However, our findings seemed to contradict the results of these studies. In the current study, a genetic liability to high levels of IL-6 and TNFa was not statistically associated with CKD and kidney function. Apart from IL-1b and IL-18, we also noted other suggestive causal associations between systemic inflammatory regulators and CKD or kidney function, including bNGF, GROa, IL-7 and SCGFb with CKD, MIP1b and TRAIL with BUN and IL-2ra, IL-4, MIG and MIP1b with eGFRcrea. These relationships are new and the paucity of relevant research may hinder us from understanding these associations. Therefore, further exploration of these associations in human observational studies or trials or animal experimental studies is imperative.

Somewhat intriguingly, despite no statistically significant causal relationships being detected in the forward MR analyses, we identified 6 statistically significant and 11 suggestive associations illustrating the MR effects of CKD or kidney function on several systemic inflammatory regulators in the reverse MR analyses. Those statistically significant causal associations included CKD, eGFRcys with GCSF, CKD, eGFRcrea with SCF, eGFRcys with IFNg and eGFRcys with VEGF. As Ferrucci L et al. [35] concluded, age-related increase in the levels of pro-inflammatory markers in blood and tissues is highly prevalent in elderly individuals with multiple chronic disease (i.e., CKD and cardiovascular diseases (CVD)). This was in consistent with our findings. It is well established that GCSF is a type of growth factor that stimulates the bone marrow to generate neutrophils and release them into bloodstream and elevated peripheral blood neutrophil and monocyte counts are an independent risk factor for CVD [37, 38]. Accordingly, the high prevalence of CVD in patients with CKD or ESRD may be due to the impact of increased GCSF derived from the impaired kidney exerted on cardiovascular system. Contrary to GCSF, higher SCF level seemed to be associated with less frequent cardiovascular deaths according to the study conducted by Rossignol P et al. [39]. Genetically determined CKD was related to the concentration of IL-17 (IVW OR = 1.134; 95% CI, 1.022–1.258; *P* = 0.025), which was a pro-inflammatory cytokine secreted by a distinct type of T cells, T helper cells and certain other lymphocytes and mainly involved in inflammatory and autoimmune diseases, such as psoriatic arthritis, ankylosing spondylitis and psoriasis [36, 40]. Mehrotra et al. [41] suggested that IL-17 secretion could potentially jeopardize renal function via the recruitment of neutrophils in rats and this may be one of the numerous mechanisms during acute kidney injury (AKI)-to-CKD transition [41]. Moreover, several previous reports elucidated that kidney function impairment was related to increased level of IL-17 [42–45]. Building on these data, we were inclined to believe that there may be a negative feedback loop between serum IL-17 and kidney function decline. In addition, it was reported that therapeutics targeting IL-17 had the potential to reduce CVD progression [37].

Our study is not devoid of limitations. First, to yield robust and reliable MR estimates, effective and strong IVs are necessary. Pleiotropy refers to the phenomenon where a single genetic variant influences multiple phenotypic traits or outcomes in MR analysis. If a genetic variant affects the outcome through pathways other than the exposure, it can introduce bias into the causal estimates, leading to incorrect conclusions about the relationship between the exposure and outcome. Although the results of MR-PRESSO and MR-Egger intercept test in this study did not indicate the obvious presence of horizontal and directional pleiotropy and confounders, we cannot yet exclude the possibility completely, which may impact the reliability of MR analysis results. Second, despite selecting strong IVs with F statistics larger than the frequently utilized value of 10, it seemed that information from a larger sample size would yield a more precise evaluation regarding the genetic influences on exposures in light of a relatively small fraction of the total variance in some systemic inflammatory regulators explaining the relationships between IVs and several exposures. Third, the samples of selected data for exposures and outcomes were mainly from European populations, thus it remains unclear whether our findings are still reliable when extrapolating them to individuals of non-European descent. Finally, MR provides a significant method for investigating the MR effects of exposures on outcomes; however, considering that risk proxy SNPs reflect lifetime exposure factors rather than a specific therapeutic intervention within a short period of time, the MR effect estimates in the present study should be treated cautiously when applied to disease management and patient counseling. To further confirm the MR effects of exposures on outcomes, randomized controlled trials of preventive interventions for short-term interventions are desirable.

## Conclusions

In conclusion, using large-scale GWAS data, we performed bidirectional two-sample MR analyses to infer the underlying causal relationships between systemic inflammatory regulators and CKD and kidney function. Our findings support causal links between systemic inflammatory regulators and CKD or kidney function both in the forward and reverse MR analyses. This may provide additional insights into the etiology and progression mechanism of CKD and contribute to making prevention policies.

### Electronic supplementary material

Below is the link to the electronic supplementary material.

Supplementary Material 1

## Data Availability

Summary data on the 41 systemic inflammatory regulators were available at https://www.ebi.ac.uk/gwas/downloads/summary-statistics. Summary data on CKD and kidney fucntion were downloaded from the CKDGen consortium at https://ckdgen.imbi.uni-freiburg.de/.
